# Serum-Derived Bovine Immunoglobulin Promotes Barrier Integrity and Lowers Inflammation for 24 Human Adults Ex Vivo

**DOI:** 10.3390/nu16111585

**Published:** 2024-05-23

**Authors:** Pieter Van den Abbeele, Charlotte N. Kunkler, Jonas Poppe, Alexis Rose, Ingmar A. J. van Hengel, Aurélien Baudot, Christopher D. Warner

**Affiliations:** 1 Cryptobiotix SA, Technologiepark-Zwijnaarde 82, 9052 Ghent, Belgium; jonas.poppe@cryptobiotix.eu (J.P.); aurelien.baudot@cryptobiotix.eu (A.B.); 2 Proliant Health & Biologicals, LLC., Ankeny, IA 50021, USA; charlotte.kunkler@phb1.com (C.N.K.); alexis.rose@phb1.com (A.R.); christopher.warner@phb1.com (C.D.W.)

**Keywords:** systemic intestinal fermentation research (SIFR), lipopolysaccharides (LPS), short-chain fatty acid (SCFA), branched-chain fatty acids (bCFA), *Bacteroides vulgatus*, *Coprococcus comes*, *Bifidobacterium adolescentis*, *Anaerostipes hadrus*, *Faecalibacterium prausnitzii*, linear mixed model

## Abstract

Serum-derived bovine immunoglobulin (SBI) prevents translocation and inflammation via direct binding of microbial components. Recently, SBI also displayed potential benefits through gut microbiome modulation. To confirm and expand upon these preliminary findings, SBI digestion and colonic fermentation were investigated using the clinically predictive ex vivo SIFR^®^ technology (for 24 human adults) that was, for the first time, combined with host cells (epithelial/immune (Caco-2/THP-1) cells). SBI (human equivalent dose (HED) = 2 and 5 g/day) and the reference prebiotic inulin (IN; HED = 2 g/day) significantly promoted gut barrier integrity and did so more profoundly than a dietary protein (DP), especially upon LPS-induced inflammation. SBI also specifically lowered inflammatory markers (TNF-α and CXCL10). SBI and IN both enhanced SCFA (acetate/propionate/butyrate) via specific gut microbes, while SBI specifically stimulated valerate/bCFA and indole-3-propionic acid (health-promoting tryptophan metabolite). Finally, owing to the high-powered cohort (n = 24), treatment effects could be stratified based on initial microbiota composition: IN exclusively stimulated (acetate/non-gas producing) *Bifidobacteriaceae* for subjects classifying as *Bacteroides*/*Firmicutes*-enterotype donors, coinciding with high acetate/low gas production and thus likely better tolerability of IN. Altogether, this study strongly suggests gut microbiome modulation as a mechanism by which SBI promotes health. Moreover, the SIFR^®^ technology was shown to be a powerful tool to stratify treatment responses and support future personalized nutrition approaches.

## 1. Introduction

Oral immunoglobulins contribute to gut homeostasis by binding to microbial components, thus preventing inflammatory responses [1,2]. Serum-derived bovine immunoglobulin isolate (SBI) is a specific concentrated serum protein fraction rich in such immunoglobulins, particularly IgG. Besides beneficial effects in an animal colitis model [3], SBI has been shown to improve intestinal barrier integrity and decrease inflammation in HIV-infected subjects on suppressive antiretroviral therapy with chronic diarrhea [4]. The mechanism by which SBI improves health involves binding of IgG to conserved microbial and viral antigens (thus preventing translocation of bacterial components over the epithelium and mitigating inflammation [2,5]), while Petschow et al. proposed three additional mechanisms: (i) maintenance of gastrointestinal immune homeostasis, (ii) preservation of gut barrier function and (iii) beneficial impact on the gut microbiome [6,7]. A recent ex vivo study indeed revealed that SBI is partially indigestible and could exert benefits via gut microbiome modulation [8]. The gut microbiome has indeed been linked to health via the production of a myriad of metabolites [9]. The gut microbiome-modulating potential of SBI and the potential link with health benefits is still preliminary given the limitations of the aforementioned ex vivo study: (i) no comparators to benchmark the microbiome-modulation capacity of SBI, (ii) a rather small number of test subjects (n = 6) and (iii) no assessment of effects on host cells.

Clinical trials are required to demonstrate health benefits, but due to large variations in gut microbiome composition [10], understanding gut microbiome modulation is challenging in vivo. In addition, microbial metabolites are hard to trace, given their rapid absorption in vivo [11,12]. Since its introduction [13], the high-throughput, bioreactor-based, ex vivo SIFR^®^ technology has proven to be a useful tool to decipher changes in the gut microbiome. In contrast to in vivo studies, it minimizes variability and enables insights into metabolite production. A strength (over legacy in vitro technologies) is the accurate preservation of in vivo-derived microbiota in the laboratory, while the high throughput enables the inclusion of a large number of biological replicates, which is required to address interpersonal differences [10]. Altogether, this technique enables the successful translation of laboratory findings on gut microbiome modulation (down to species level) to clinical observations [13]. Applications of the SIFR^®^ technology meanwhile range from characterizing the microbiome-modulating potential of probiotics [14], prebiotics [13,15], sweeteners [16], development of synbiotics [14], age-specific ingredients [17] and investigation of fiber specificity [15], along with studying microbial diversity (using novel indices) [18]. So far, no studies have been performed to explore how gut microbiome modulation in SIFR^®^ technology-derived samples translates to effects on intestinal barrier integrity and/or immune functioning.

The present study aimed to assess the gut microbiome-modulating potential of SBI using the ex vivo SIFR^®^ technology (for 24 human adults). Further, potential effects on gut barrier integrity and immune modulation were investigated using a coculture of epithelial/immune (Caco-2/THP-1) cells that was, for the first time, combined with the recently introduced, clinically validated SIFR^®^ technology. To benchmark effects with SBI (tested at a human equivalent dose (HED) of 2 and 5 g/day), two comparators were tested in parallel: a reference dietary protein (DP; milk proteins also tested at a HED of 2 and 5 g/day; rationale for inclusion is that like SBI, DP is a protein which enables testing whether effects are common amongst proteins are unique to SBI) and the reference prebiotic inulin (IN; tested at a HED of 2 g/day; rationale for inclusion; inulin is known to potently impact the gut microbiome and host health, thus allowing for benchmarking potential beneficial effects of SBI [19]). A unique aspect of the study was that the high-powered cohort (n = 24) enabled stratifying treatment responses based on initial microbiota composition, thus supporting future personalized nutrition.

## 2. Materials and Methods

### 2.1. Test Compounds

SBI was provided by Proliant Health & Biologicals LLC. (Ankeny, IA, USA). SBI contains 93.0% protein, 3.0% moisture and 1.5% ash. The protein fraction consists of >50% IgG, with the remainder consisting of serum proteins such as albumin, transferrin and α2-macroglobulin. Milk-derived proteins were included as a reference for dietary protein (DP) intake. Like Toffolon et al. (2021) [20], a ratio of whey protein isolate (unflavoured, Myprotein) over casein (Carl Roth 8569) of 4:1 was tested. Another comparator was the reference prebiotic IN from chicory origin (Sigma I2255). IN is a polymer of β(2,1)-bond-linked fructose residues with a chain-terminating glucose with a fructose/glucose ratio of 20:1.

### 2.2. Experimental Design, Timeline and Analysis

**Figure 1:**
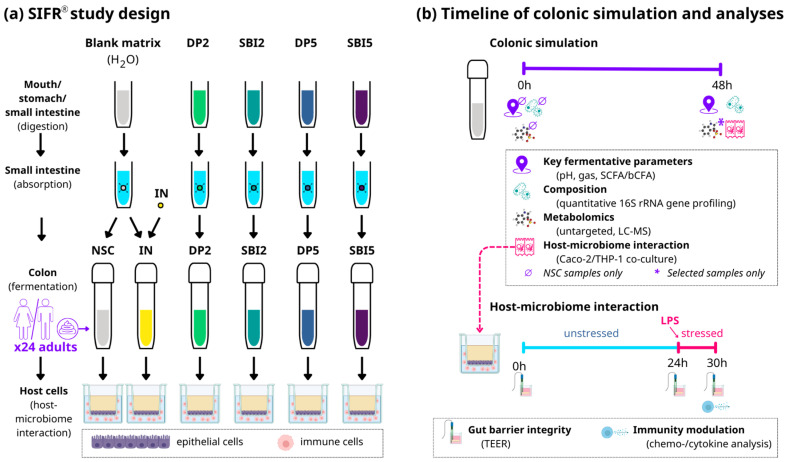
**The impact of SBI, the reference protein DP (both tested at HED = 2 and 5 g/day) and the reference prebiotic IN (tested at HED = 2 g/day) was assessed on the gut microbiome, gut barrier integrity and immune modulation using the ex vivo SIFR^®^ technology for 24 human adults.** (**a**) Study design of full gastrointestinal simulation and host–microbiome interaction assay along with (**b**) timeline and analysis. SIFR^®^ = systemic intestinal fermentation research; NSC = no-substrate control; IN = inulin; DP = dietary protein; SBI = serum-derived bovine immunoglobulin; SCFA = short-chain fatty acids; bCFA = branched-chain fatty acids; LC-MS = liquid chromatography–mass spectrometry, HED = human equivalent dose.

Upper gastrointestinal digestion/absorption and colonic fermentation were investigated using the SIFR^®^ technology as recently described [8,13]. This allowed for studying the gut microbiome modulation potential of the various test products. Six study arms were tested for 24 human adults: an unsupplemented control (no substrate control, NSC), IN (HED = 2 g/day), SBI (HED = 2 and 5 g/day; SBI2 and SBI5) and DP (HED = 2 and 5 g/day; DP2 and DP5) (Figure 1a). SBI was tested at doses equivalent to (i) 5 g/day, simulating its intake as part of a medical food (EnteraGam^®^; https://enteragam.com/, last accessed on 2 May 2024) and (ii) 2 g/day based upon its inclusion as a dietary supplement. These doses also aligned with doses that were tested in clinical trials that revealed the beneficial effects of SBI on barrier integrity and immune modulation [4]. While DP was tested at the exact same doses as SBI, IN was only tested at 2 g/day (given that IN fully reaches the colon, while SBI is partially digested and absorbed along the colon).

Briefly, test products (or distilled H_2_O for NSC/IN) were subjected to oral, gastric and small intestinal digestion according to the INFOGEST 2.0 method [21]. To ensure compatibility with the colonic incubations, modifications were implemented, such as the removal of oxygen and simulation of small intestinal absorption [8]. At the start of the colonic incubations, individual fecal samples were processed in a bioreactor management device (Cryptobiotix, Ghent, Belgium) [13]. Each bioreactor contained 5 mL of a blend of small intestine-derived suspension, nutritional medium (M0017, Cryptobiotix, Ghent, Belgium) and a fecal inoculum from a single donor. Bioreactors were sealed individually and rendered anaerobic. After preparation, bioreactors were incubated under continuous agitation (140 rpm) at 37 °C for 48 h (MaxQ 6000, Thermo Scientific, Merelbeke, Belgium). Upon gas pressure measurement, samples were collected at 0h and 48h for measurement of key fermentation parameters (pH, gas production and SCFA analysis), microbial composition and host–microbiome interactions (Figure 1b)**.**

Untargeted metabolite profiling was only applied on a subset of study arms (NSC/IN/SBI5) and donors (6/9/12/15/20/22) to obtain exploratory insights into metabolite production beyond key fermentation parameters. Control samples (NSC) were run in technical triplicate for each of the 24 adults and monitored for pH, gas and SCFA production, with coefficients of variation below 3%, confirming the high technical reproducibility of the SIFR^®^ technology [13]. Fresh fecal samples were collected according to a procedure approved by the Ethics Committee of the University Hospital Ghent (reference number BC-09977). This procedure involved participants signing an informed consent and donating their fecal samples for the study. The selection criteria for human adults were no antibiotic use in the past 3 months, no gastrointestinal disorders (cancer, ulcers, IBD), non-smoking, alcohol consumption <3 units/d, and 25–65 years of age. Twelve male and twelve female human adults were sourced with an average age of 37.6 (±10.0 years).

### 2.3. Host–Microbiome Interaction Assay

To understand how the modulation of the gut microbiome had downstream effects on the host (as assessed via the SIFR^®^ technology), a coculture experiment with epithelial cells (human adenocarcinoma Caco-2 cell line) and immune cells (human acute monocytic leukemia THP1 cell line, differentiated to activated macrophages by a PMA treatment) was implemented, as published before [22]. Briefly, upon differentiation of Caco-2 and THP1-cells over 14 and 2 days, respectively, a model gut wall was created by covering immune cells with an epithelial layer in a permeable well insert (Figure 1a). SIFR^®^-derived colonic samples were centrifuged (5′ at 9000× *g*) and filtered (0.22 µm) prior to being used in the assay. The host–microbiome interaction assay consisted of two phases (Figure 1b): (i) 24 h treatment during which colonic samples were applied on the apical side of epithelial cells (diluted in cell medium) to evaluate their impact on gut barrier integrity under unstressed conditions, and (ii) a subsequent additional 6h incubation in the presence of 500 ng/mL lipopolysaccharide (LPS) to evaluate effects on barrier integrity under stressed conditions, along with potential immunomodulatory effects (at 30 h). Transepithelial electrical resistance (TEER) is a widely accepted method to measure gut barrier integrity [23] and was measured before administration of colonic samples (0 h), at 24 h (before LPS addition) and at 30 h (6 h after LPS addition). At each time point, TEER values of treatments (Ω·cm^2^) were normalized compared to the NSC (within each of the 24 test subjects). Immune effects were studied via cytokine/chemokine production using a Multiplex Luminex^®^ Assay kit on the MAGPix^®^ analyzer (IL-6, CXCL10, IL-10, IL-1β, TNF-α) or ELISA (IL-8).

### 2.4. Key Fermentation Parameters

SCFA (acetate, propionate, butyrate and valerate) and branched-chain fatty acids (bCFA; the sum of isobutyrate, isocaproate and isovalerate) were determined via gas chromatography with flame ionization detection, upon diethyl ether extraction, as previously described [15]. pH was measured using an electrode (Hannah Instruments Edge HI2002, Temse, Belgium).

### 2.5. Taxonomic Microbiota Analysis by Quantitative 16S rRNA Gene Profiling

DNA was extracted via the SPINeasy DNA Kit for Soil (MP Biomedicals, Eschwege, Germany), according to the manufacturer’s instructions. Subsequently, library preparation and sequencing were performed on an Illumina MiSeq platform with v3 chemistry. The 16S rRNA gene V3–V4 hypervariable regions were amplified using primers 341F (50 -CCT ACG GGN GGC WGC AG-30) and 785Rmod (50 -GAC TAC HVG GGT ATC TAA KCC-30). Pre-processing and OTU (operational taxonomic unit) picking from amplicons was performed with Mothur v1.35.1 [24], including annotation of representative sequences with NCBI blast v2.10.0 [25]. The results were analyzed at phylum, family and OTU levels.

To determine total counts, samples were diluted in anaerobic phosphate-buffered saline (PBS), followed by cell staining with SYTO 16 at a final concentration of 1 µM, and counted via a BD FACS Verse flow cytometer (BD, Erembodegem, Belgium). Data were analyzed using FlowJo, version 10.8.1. Quantitative insights were obtained by correcting proportions (%; 16S rRNA gene profiling) with total counts (cells/mL; flow cytometry), resulting in estimated cells/mL of different taxa.

### 2.6. Untargeted Metabolite Profiling

Liquid chromatography–mass spectrometry (LC-MS/MS) analysis was carried out to obtain insights into microbial metabolite production well beyond SCFA/bCFA. The analysis was performed on a Thermo Scientific Vanquish LC coupled to Thermo Q Exactive HF MS (Thermo Scientific, Waltham, MA, USA) using an electrospray ionization source. The analysis was performed both in negative and positive ionization modes. The UPLC was performed by applying a slightly modified version of the protocol described by Doneanu et al. [26]. Peak areas were extracted using Compound Discoverer 3.1 (Thermo Scientific), along with a manual extraction based on an in-house library using Skyline 21.1 (MacCoss Lab Software) [27]. Identification of compounds was performed at different levels: level 1 (retention times (compared against in-house authentic standards), accurate mass (with an accepted deviation of 3 ppm), and MS/MS spectra)), level 2a (retention times and accurate mass), level 2b (accurate mass and MS/MS spectra), and level 3 (accurate mass alone). Technical variability was confirmed by running a QC sample (pooled sample of all samples) every six samples. Coefficients of variation for these QC samples were, on average, 5.6% for level 1-annotated metabolites, confirming the high technical reproducibility of the method.

### 2.7. Data Analysis

Except for the principal component (PCA) analysis, which was performed with GraphPad Prism (v9.3.1; www.graphpad.com; accessed on 20 November 2023), other univariate and multivariate analyses were performed using R (version 4.2.2; www.r-project.org; accessed on 5 February 2024). These analyses include violin plots, bar charts and heat maps. While violin plots and bar charts present the actual values, heat maps present log_2_-transformed fold changes for the different treatments compared to the parallel control arm (NSC). This way, when a metabolite or microbial taxa is increased by a given treatment, a positive value is displayed, while negative values reflect a decrease. The significance of the effects of supplementation was assessed via repeated measures ANOVA analyses (based on paired testing among the 24 human adults), with *p*-value correction according to Benjamini–Hochberg [28]. For the analysis of microbial composition, three measures were taken. First, the statistical analysis was performed on log_10_-transformed values. Second, a value of a given taxonomic group below the limit of detection (LOD) was considered equal to the overall LOD, as described recently [13]. Third, a threshold was set to retain the 100 most abundant OTUs in the analysis to avoid major *p*-value corrections. Regularized Canonical Correlation Analysis (rCCA) was executed using the mixOmics package with the shrinkage method for the estimation of penalization parameters (version 6.20.3) [29].

Finally, to assess whether initial fecal microbiota composition impacted treatment outcomes, a linear mixed model (LMM) approach was applied, similar to the recently published by Vandeputte et al. (2017) [30]. Briefly, the 24 human adult microbiota were classified as Prevotella (P) or Bacteroides/Firmicutes enterotypes (BF). Further, the LMM consisted of the following terms: impact of P vs. BF on NSC values (=1 term), individual treatment effect vs. NSC (IN, DP2, DP5, SBI2, SBI5 = 5 terms), and 5 terms to scope for interaction between P vs. BF donors and treatment effects. The latter term enabled assessing whether different microbial compositions at baseline impacted treatment effects.

## 3. Results

### 3.1. The Study Cohort Covered a Relevant Spectrum of Interpersonal Differences in Microbiota Composition

**Figure 2:**
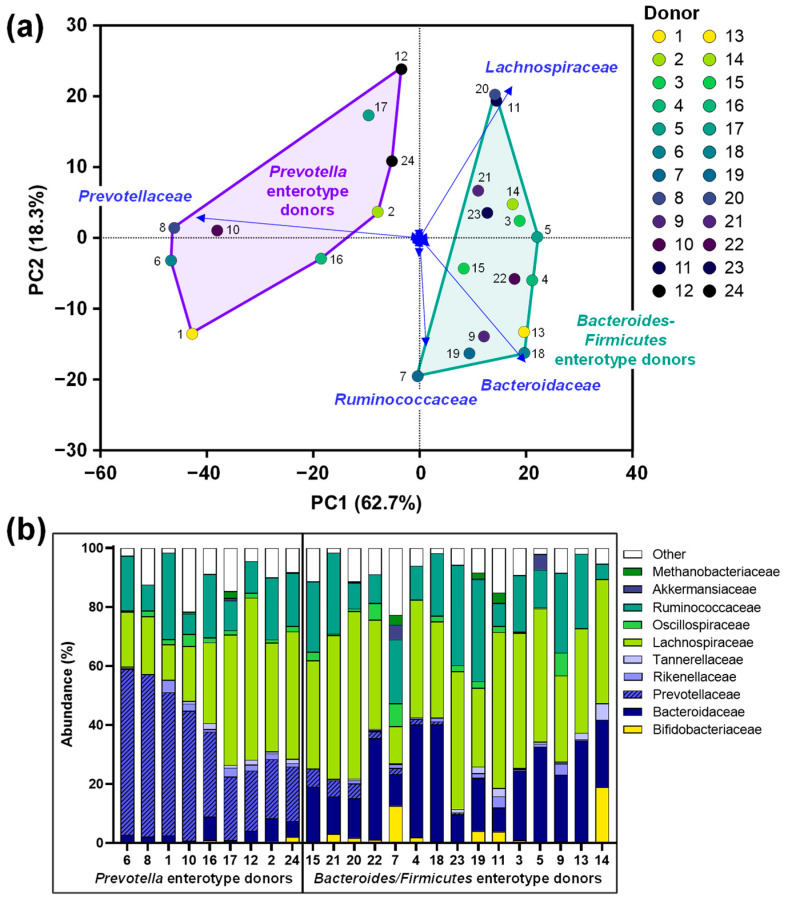
The fecal microbiota of 24 human adult donors covered clinically relevant interpersonal differences: (**a**) PCA based on centered abundances at the family level (%) demonstrating the variation across the fecal microbiota. (**b**) Abundances (%) of the key families. PCA = principal component analysis.

The fecal microbiota of the 24 human adults displayed marked interpersonal differences, mostly due to different levels of *Prevotellaceae*, *Bacteroidaceae* and *Lachnospiraceae/Ruminococcaceae* (Figure 2a,b). This observation is in line with the established classification of the human gut microbiota according to the Prevotella, Bacteroides and Firmicutes enterotypes [31]: 9 human adults classified as Prevotella enterotype donors (Figure 2, left), 15 classified as Bacteroides/Firmicutes enterotype donors (Figure 2, right).

### 3.2. SBI Promoted Gut Barrier Integrity While Suppressing Pro-Inflammatory Markers

Upon oral, gastric, small intestinal and colonic incubation, samples of the various study arms were added to the host–microbiome interaction assay to assess effects on gut barrier integrity and immune modulation.

**Figure 3:**
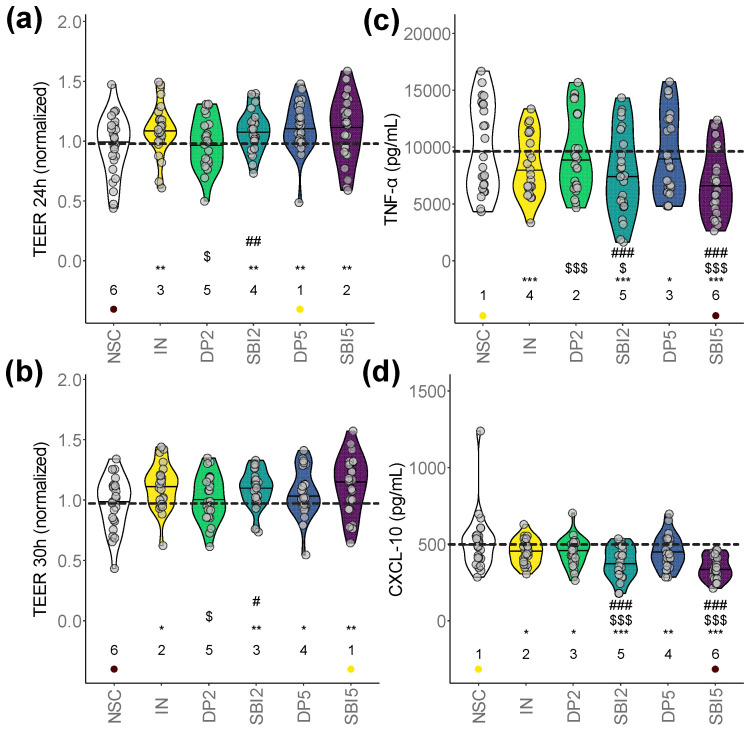
**SBI promoted gut barrier integrity while suppressing pro-inflammatory markers.** The host–microbiome interaction assay assessed gut barrier integrity (TEER, normalized to 0 h) under (**a**) unstressed (24 h) and (**b**) stressed conditions (30 h). Immune modulation was assessed via the (**c**) pro-inflammatory cytokine TNF-α and (**d**) chemokine CXCL-10. Statistical differences with the unsupplemented control NSC are visualized via * (0.01 < *p*_adjusted_ < 0.05), ** (0.001 < *p*_adjusted_ < 0.01) or *** (*p*_adjusted_ < 0.001); ‘$/$$/$$$’ indicate differences with the reference prebiotic IN, and ‘#/##/###’ indicate differences between corresponding doses of SBI and the reference protein DP (0.01–0.05/0.001–0.01/<0.001). There were no significant differences between SBI2 and SBI5. Ranks of average values per study arm are shown, with the lowest/highest values being indicated in purple/yellow, respectively. The dashed line indicates the average value of a given parameter for the NSC.

First, TEER was measured to evaluate the integrity of the epithelial barrier (higher values reflect an increased barrier integrity). All products (except DP2) significantly increased barrier integrity compared to NSC, both under unstressed (Figure 3a) and stressed conditions (Figure 3b). Under the unstressed conditions, the strongest increases were noted for IN/DP5/SBI5, while under stressed conditions, IN and SBI (both doses) most strongly improved barrier integrity. Further, SBI2 significantly increased TEER compared to DP2 (both under stressed/unstressed conditions), with SBI5 also (non-significantly) increasing TEER compared to DP5 under stressed conditions. Thus, SBI particularly promoted gut barrier integrity under stressed conditions.

All test products (except DP2) also significantly lowered the pro-inflammatory cytokine TNF-α (Figure 3c) and inflammatory chemokine CXCL10 (Figure 3d). TNF-α/CXCL-10 levels were significantly lower for SBI2 compared to IN/DP2 and for SBI5 compared to IN/DP5, stressing that SBI most strongly suppressed both pro-inflammatory markers. In contrast to TNF-α/CXCL-10, milder effects were observed for IL-10, IL1-β, IL-6 and IL-8, amongst others, including a significant decrease in IL-6 for SBI2 (Figure S1a–d).

Overall, while the extent of the effects on barrier integrity and immune modulation was often larger for SBI5 compared to SBI2, these differences were not significant, suggesting that SBI already exerts potent effects at a HED of 2 g/day.

### 3.3. IN and SBI Stimulated Microbial Metabolite Production

**Figure 4:**
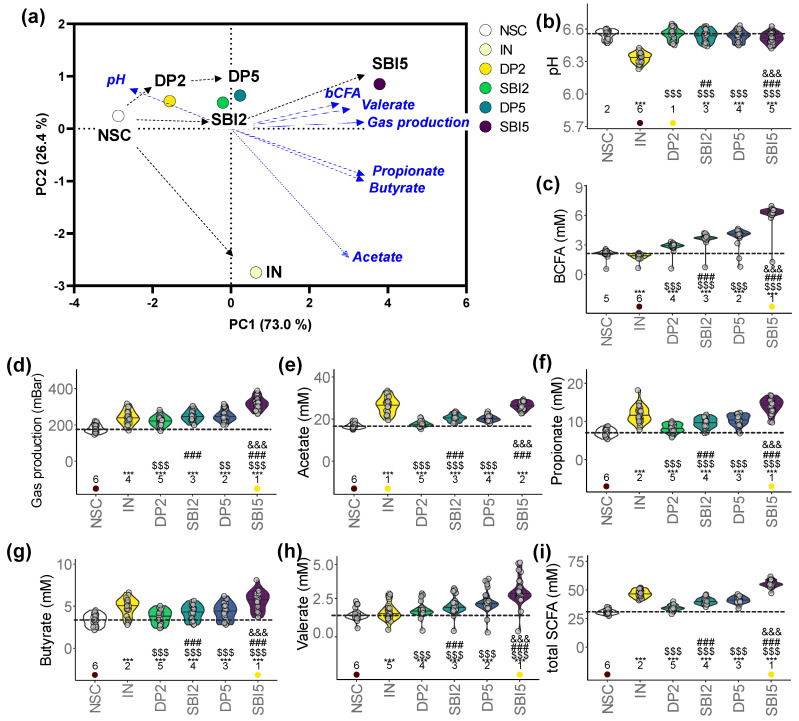
IN and particularly SBI impacted key fermentation parameters of the gut microbiome of 24 human adults as tested with the ex vivo SIFR^®^ technology. (**a**) PCA summarizing the overall impact on key fermentation parameters at the end of the colonic incubation, including (**b**) pH, (**c**) gas production, (**d**) acetate, (**e**) propionate, (**f**) butyrate, (**g**) valerate, (**h**) total SCFA and (**i**) bCFA. Statistical differences with the unsupplemented control NSC are visualized via * (0.01 < *p*_adjusted_ < 0.05), ** (0.001 < *p*_adjusted_ < 0.01) or *** (*p*_adjusted_ < 0.001); ‘$/$$/$$$’ indicate differences with the reference prebiotic IN, and ‘#/##/###’ indicate differences between corresponding doses of SBI and the reference protein DP, and ‘&/&&/&&&’ between the two doses of SBI (SBI2 and SBI5) (0.01–0.05/0.001–0.01/<0.001). Ranks of average values per study arm are shown, with the lowest/highest values being indicated in purple/yellow, respectively. The dashed line indicates the average value of a given parameter for the NSC.

The distances between the study arms in the PCA of the key fermentation parameters (pH, gas, SCFA, bCFA) revealed the following key insights (Figure 4a): (i) marked difference between IN and the protein-based test products (DP/SBI); (ii) among protein-based products, SBI exerted stronger effects compared to DP; (iii) 5 g/day of SBI/DP exerted stronger effects than the HED of 2 g/day.

A key difference between IN and the protein-based test products (DP/SBI) was that IN strongly decreased pH (Figure 4b). In addition, while IN significantly lowered bCFA production, DP- and especially SBI-based products significantly increased bCFA (Figure 4c). Further, despite differences in the extent of effects, all test products significantly increased gas production (Figure 4d) and acetate, propionate, butyrate and valerate (thus, total SCFA) (Figure 4e–i). While IN most specifically enhanced acetate, protein-based treatments more specifically increased valerate. Among protein-based products, SBI always exerted significantly stronger effects than DP, with significantly stronger effects at higher test doses. Overall, SBI5 most strongly increased propionate, butyrate, valerate (thus, total SCFA) and bCFA amongst all study arms.

**Figure 5:**
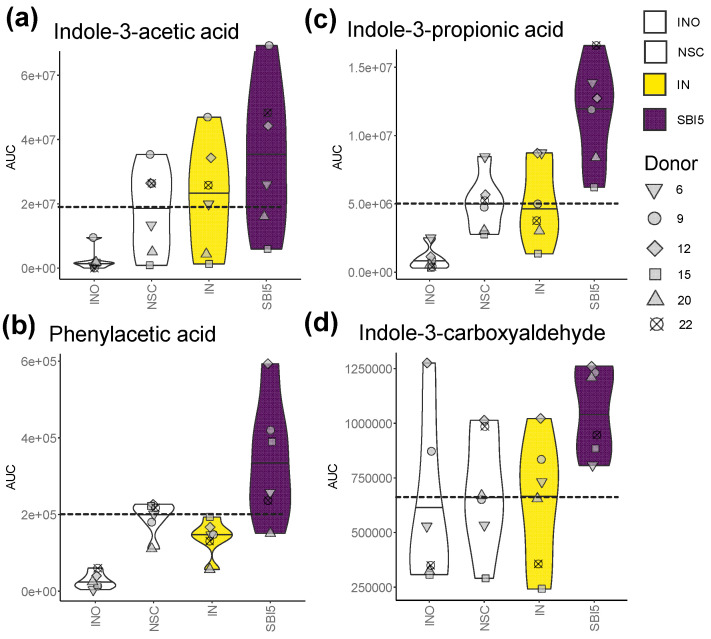
Untargeted metabolomics revealed that SBI5 impacted microbial metabolite production beyond traditionally studied key fermentation parameters. (**a**) Indole-3-acetic acid, (**b**) indole-3-propionic acid, (**c**) phenylacetic acid and (**d**) indole-3-carboxyaldehyde were analyzed at the end of the colonic incubations for a subset of study arms (NSC/IN/SBI5) and donors that covered the spectrum of microbiota composition across the cohort (6/9/12/15/20/22 (Figure 2)). The dashed line indicates the average value of a given parameter for the NSC.

Finally, to evaluate the production of metabolites beyond traditionally studied key fermentation parameters, untargeted metabolomics was implemented. This analysis was applied for a subset of the most potent study arms (NSC/IN/SBI5) and for six donors that covered the spectrum of microbiota composition (from Prevotella enterotypes (6/12) to Bacteroides enterotypes (9/22), along with microbiota in-between (15/20) (Figure 2a,b)). These results further illustrated the potential of SBI to modulate the gut microbiome activity, i.e., by significantly stimulating the production of phenylalanine/tryptophan metabolites such as indole-3-acetic acid (*p* = 0.009), phenylacetic acid (*p* = 0.045), indole-3-propionic acid (IPA) (*p* = 0.002) and indole-3-carboxyaldehyde (*p* = 0.016) (Figure 5a–d).

### 3.4. IN and SBI Each Stimulated a Specific Spectrum of Gut Microbes

A high-level observation was that all test products significantly increased bacterial cell density, with the extent of the effects increasing from DP2 < DP5 < SBI2 < IN < SBI5 (Figure S2a). A significant decrease in Shannon diversity for all products further suggested lower species evenness and, thus, a specific stimulation of gut microbes by all test products, particularly IN/SBI5 (Figure S2b).

**Figure 6:**
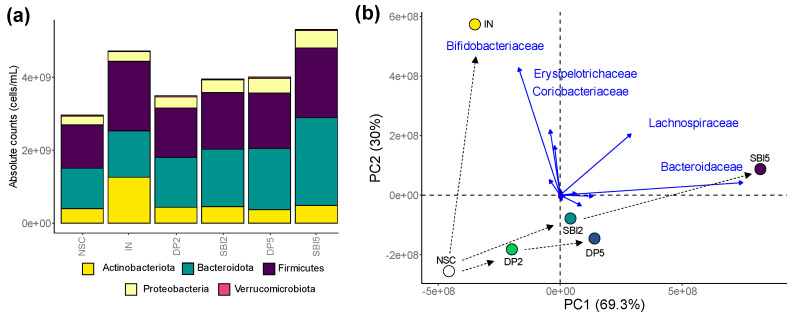
While IN enhanced *Bifidobacteriaceae* (<Actinobacteriota), SBI stimulated *Bacteroidaceae* (<Bacteroidota) and *Lachnospiraceae* (<Firmicutes) in the microbiome of human adults, as tested with the ex vivo SIFR^®^ technology. (**a**) Microbial composition (phylum level) presented as absolute values (cells/mL), obtained by multiplying proportional values (%) with total cell counts (cells/mL), after which averages across 24 test subjects within test conditions were calculated. (**b**) PCA summarizing the impact on composition (at the family level), based on average absolute values (cells/mL), with an indication of five families that most strongly contributed to the clustering.

A first visualization at the phylum level (Figure 6a), and especially the differential positioning of study arms in the exploratory analysis of microbial composition (family level), confirmed the following key findings (Figure 6b): (i) marked difference between IN and protein-based test products; (ii) SBI exerted stronger effects than DP; (iii) 5 g/day SBI/DP exerted stronger effects than a HED of 2 g/day. IN specifically enhanced *Bifidobacteriaceae/Coriobacteriaceae* (<Actinobacteriota) and *Erysipelotrichaceae* (<Firmicutes), while SBI instead stimulated *Bacteroidaceae* (<Bacteroidota) and *Lachnospiraceae* (<Firmicutes).

**Figure 7:**
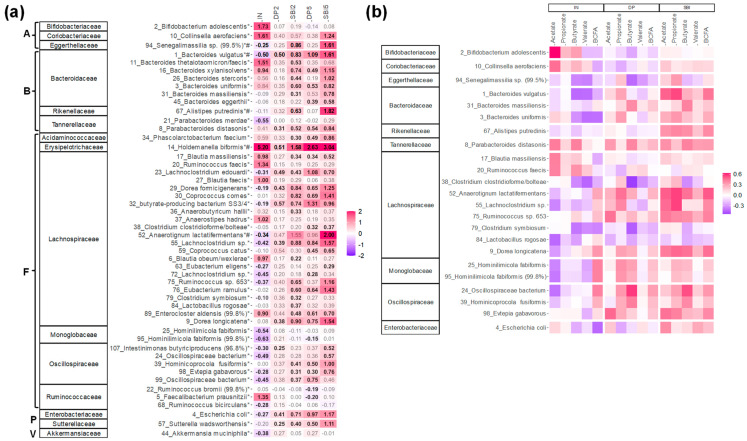
**Compared to IN, SBI enhanced a different and broader range of gut microbes for 24 human adults tested with the ex vivo SIFR^®^ technology.** (**a**) Heatmap based on OTUs that were significantly (FDR = 0.20) affected by any of the treatments, expressed as log_2_ (treatment/NSC), averaged over the 24 human adults. Values indicated in bold show significant increases (>0) or decreases (<0). Corresponding families and phyla are indicated on the left for each OTU. The phyla to which the families belong are indicated by letters (A = Actinobacteriota; B = Bacteroidota; F = Firmicutes; P = Proteobacteria; V = Verrucomicrobiota). (**b**) rCCA to highlight correlations of these OTUs with key fermentation parameters for IN, DP (dataset comprising samples of DP2/DP5) and SBI (dataset comprising samples of SBI2/SBI5). * indicates species that were significantly affected, while # indicates the species that explained most variation among treatments.

An in-depth analysis at the highest taxonomic resolution (OTU level) revealed the key contributors to the fermentation of the various test products (Figure 7a), often correlating with the production of specific metabolites (Figure 7b).

First, IN specifically increased *Bifidobacterium adolescentis* (OTU2; <*Bifidobacteriaceae*) and *Collinsella aerofaciens* (OTU10; <*Coriobacteriaceae*), both correlating with acetate production. Further, IN specifically increased *Holdemanella biformis* (OTU14; <*Erysipelotrichaceae*) along with specific members of the *Bacteroidaceae* (*B. thetaiotaomicron* (OTU11) and *B. xylanisolvens* (OTU16)), *Lachnospiraceae* (e.g., *Anaerostipes hadrus* (OTU37) and several *Blautia* species) and *Ruminococcaceae* (*Faecalibacterium prausnitzii* (OTU5)). IN also significantly decreased the range of OTUs.

The protein-derived products stimulated different taxa, with more profound effects as test doses increased. SBI5 stimulated the following taxa more strongly compared to DP5: (i) *Bacteroidaceae* (*B. vulgatus* (OTU1), besides OTUs related to *B. xylanisolvens*, *B. stercoris*, *B. uniformis*, *B. massiliensis* and *B. eggerthii*); (ii) *Tannerellaceae* (*Parabacteroides distasonis* (OTU8)); (iii) *Acidaminococcaceae* (*Phascolarctobacterium faecium* (OTU34)); (iv) *Erysipelotrichaceae* (*Holdemanella biformis* (OTU14)); (v) *Lachnospiraceae* (14 OTUs including, amongst others, two *Dorea* species (OTU9/29), two *Lachnoclostridium* species (OTU23/55), *Coprococcus comes* (OTU30), the butyrate-producing bacterium SS3/4 (OTU32) and *Anaerotignum lactatifermentans* (OTU55)); (vi) *Oscillospiraceae* (4 OTUs); (vii) *Enterobacteriaceae*; and (viii) *Sutterellaceae.* Finally, SBI exerted specific effects (not observed for DP) on *Collinsella aerofaciens* (OTU10), a *Senegalimassilia* species (OTU94: <*Eggerthellaceae*) and *Alistipes putredinis* (OTU67; <*Rikenellaceae*). For many of the aforementioned species, correlations with acetate, propionate, butyrate, valerate and/or bCFA were observed, suggesting the involvement of a broad range of species in the fermentation, particularly of SBI.

### 3.5. Stratification of Donor Responses

**Figure 8:**
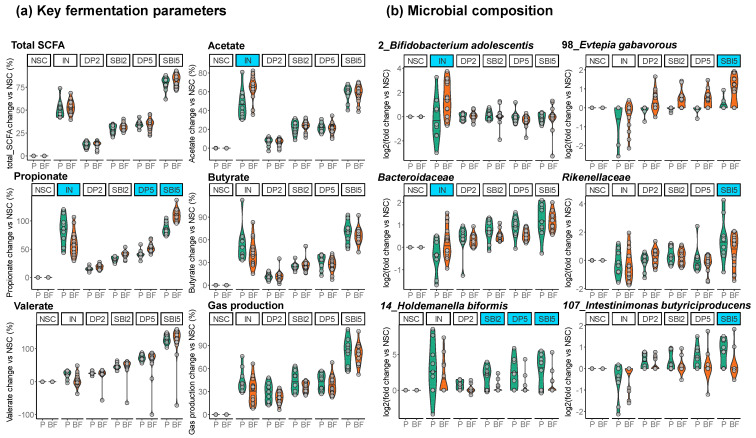
**Treatment effects of IN and SBI5 stratified based on initial fecal microbiota composition (Prevotella enterotype (P, green) and Bacteroides–Firmicutes enterotype (BF, orange) donors).** (**a**) Key fermentation parameters (change (%) compared to unsupplemented control NSC) and (**b**) microbial composition (log_2_ (treatment/NSC)). Treatments for which initial microbiota composition significantly impacted treatment effects are highlighted by blue shading.

To assess whether initial fecal microbiota composition impacted treatment outcomes, a linear mixed model (LMM) approach was applied. Besides confirming a significant contribution of the term treatment (*p* = 0.001), this analysis also revealed a significant interaction between treatment and baseline microbiota composition (*p* = 0.002). In other words, donors with a microbiota classified as Prevotella (P) responded differently to the treatments compared to donors with a microbiota classified as Bacteroides/Firmicutes enterotype (BF). While Table S1 reports model parameters for each endpoint, Figure 8 shows the treatment effects on key fermentation parameters and microbial composition (six selected features, each), as stratified based on initial fecal microbiota composition.

The first key finding was that the stimulation of *Bifidobacterium adolescentis* (OTU2) by IN was specifically observed for BF-enterotype donors, coinciding with significantly more acetate production. In contrast, the microbiota of P-enterotype donors produced more propionate/butyrate/gases upon IN treatment without such stimulation of *B. adolescentis*.

SBI5 fermentations showed less variation due to enterotype than IN. Despite these consistent effects, SBI5 preferentially stimulated *Rikenellaceae, Holdemanella biformis* (OTU14), and *Intestinimonas butyriciproducens* (OTU107) for P-enterotype donors while favoring *Evtepia gabavorous* (OTU98) in BF-enterotype donors.

## 4. Discussion

The high throughput of the ex vivo SIFR^®^ technology allowed for testing the impact of SBI and comparators for 24 human adults. Large interpersonal differences among human adults [10] make obtaining biorelevant results from preclinical gut microbiome studies challenging. By increasing the number of donors compared to the previous study (n = 6) [8], the ex vivo SIFR^®^ technology was able to overcome this challenge, resulting in a greater number of statistically significant observations. Overall, SBI was shown to modulate both metabolic activity and composition of the gut microbiome, leading to improved gut barrier integrity and suppressed pro-inflammatory responses. SBI exhibited either unique effects or largely exceeded those of the reference dietary protein, likely due to the lower digestibility of SBI and, therefore, increased abundance in the colon. While whey protein (a primary component of DP) is easily digestible [20], IgG (a primary component of SBI) has been detected in fecal samples [32]. SBI’s primary mechanism of action is preventing translocation and inflammation via direct binding of microbial antigens. However, this study highlights a prospective secondary mechanism of action that impacts host health through the microbiome by requiring SBI to only reach the colon rather than have a full antigen-binding function.

A key finding was that SBI improved barrier integrity (Figure 3a,b). This finding is in line with the findings of Utay et al. (2019), who demonstrated that SBI intake (by HIV-infected subjects on suppressive antiretroviral therapy with chronic diarrhea) improved intestinal barrier integrity, as established by lower levels of intestinal permeability biomarkers (intestinal fatty acid binding protein [I-FABP] and zonulin) [4]. Further, the observed improvement of barrier integrity with IN was in line with previous findings in both healthy [33] and disease-mimicking cell models [34]. As reviewed by Chelakkot et al. (2018) [35], the epithelial cell layer of the gut wall performs a pivotal role as the first physical barrier against external factors. A compromised barrier integrity (‘leaky gut’) contributes to many pathological conditions, including, amongst others, inflammatory bowel disease, obesity, and metabolic disorders. The improvement of the gut barrier suggests a dual mechanism of the health-promoting actions of SBI and upholds the understood mechanism of IN.

Moreover, SBI significantly reduced pro-inflammatory markers TNF-α, CXCL10, and IL-6 (SBI2 only, Figure 3c,d and Figure S1). TNF-α is a driver of intestinal inflammation and weakens epithelial barrier integrity by inducing apoptosis in intestinal epithelial cells [36], while CXCL10, an ‘inflammatory’ chemokine, is known to mediate immune responses through the activation and recruitment of leukocytes such as T cells, eosinophils, monocytes and NK cells [37]. This anti-inflammatory effect of SBI was again in line with the findings of Utay et al. (2019), who demonstrated that SBI lowered pro-inflammatory responses established by lower levels of IL-6 [4]. In contrast to barrier integrity, the anti-inflammatory effects were uniquely observed for SBI. This evidence validates the predictivity of the Caco-2/THP-1 assay, implemented on SIFR^®^ samples, for intestinal permeability and immunomodulation outcomes in vivo.

SBI potently modulated the human adult gut microbiome, both in terms of microbial composition and metabolic activity. First, SBI stimulated the production of acetate, propionate and butyrate (Figure 4), SCFA that have each been related with particular health benefits as reviewed by Rivière et al. [38], including, amongst others, anti-inflammatory and barrier integrity-promoting effects. Further, in terms of compositional changes, the current study confirmed key findings of a recent preliminary study [8], such as stimulation of acetate/propionate-producing *Bacteroides vulgatus* [39] along with *Lachnospiraceae* members (e.g., *Dorea* spp., *Lachnoclostridium* spp. and the butyrate-producing bacterium SS3/4) (Figure 7). By increasing the number of donors from 6 (preliminary study) to 24 (current study), the translational power of the design greatly increased and exposed a series of additional species involved in SBI fermentation, amongst others, multiple *Bacteroides* species and also lesser-known species with recently uncovered health-promoting potential, such as *Holdemanella biformis* [40], *Parabacteroides distasonis* [41,42] and the succinate-consuming, propionate-producing *Phascolarctobacterium faecium* [43]. The stimulation of the butyrate-producing *Coprococcus comes* by SBI was of particular interest given the concomitant increase in indole-3-propionic acid (IPA) (Figure 5). *Coprococcus* species have indeed been linked with increased levels of IPA [44], a deamination product of tryptophan metabolism. A recent study demonstrated that IPA helps maintain intestinal epithelium homeostasis, leading to a reduction in plasma endotoxin levels and pro-inflammatory cytokines [45]. The increase across the class of tryptophan catabolites is worth noting, as indole metabolites such as indole-3-acetic acid and indole-3-carboxyaldehyde have been shown to activate the aryl hydrocarbon receptor, which reduces pro-inflammatory cytokines such as TNF-α [46] and increases gut barrier integrity through tight junctions [47]. These beneficial effects are thus in line with the beneficial effects observed for SBI during the current study. While the reference prebiotic IN stimulated SCFA production, the effect size on individual SCFAs was distinct from protein-derived test products (Figure 4). The product-specific effect of IN was further corroborated by the absence of effects of IN on IPA and the very different impact on microbial composition (Figure 5). Indeed, unlike SBI, and in line with clinical studies with IN [19,30], IN specifically increased *Bifidobacterium adolescentis* (correlating with acetate production), and the butyrate-producing *Anaerostipes hadrus* and *Faecalibacterium prausnitzii* (Figure 7). While overall exerting beneficial effects on barrier integrity, the underlying microbiome modulation was highly different for IN compared to SBI.

The large number of test subjects included in the current study enabled stratifying treatment responses based on the initial microbiota composition of the 24 adults (Figure 2). Each donor was classified as either Prevotella or Bacteroides/Firmicutes-enterotype, in line with the established concept of enterotypes [31]. SBI proved to have comparable effects regardless of donor enterotype (Figure 8). However, bifidogenic effects of IN were exclusively observed for Bacteroides/Firmicutes-enterotype donors. Unlike many other gut microbes, *Bifidobacterium* species do not produce gasses and, due to a spectrum of modes of action, have been related to human health [48,49]. While the bifidogenic effect of IN has been demonstrated before at the population level [19,30], recent studies highlighted a low specificity of IN when zooming in on individual test subjects [15,50,51], implying that while IN might lead to bifidogenic effects for some adults, such effects might not be observed for others, potentially relating with high gas production and low tolerability for such individuals [52]. Despite the decades-long investigation of IN fermentation, the present study is the first to report that treatment effects of IN can be stratified based on initial microbiota signatures (likely due to the unique study design combining a new-generation incubation strategy with multi-omics analysis). Moreover, the stratification of treatment responses to IN is likely clinically relevant given that it could predict a high gas production and warn of potential adverse events (i.e., for Prevotella-enterotype donors). Nevertheless, this study is preliminary and requires further validation through future clinical studies.

Finally, the human-derived cell lines used to simulate epithelial (Caco-2) and immune cells (THP-1) are originally derived from cancer patients (not from healthy subjects). The usefulness and limitations of Caco-2 cells have been reviewed by Sun et al. (2008) [53]. These authors pointed out the biorelevance of Caco-2 cells given how they differentiate to a confluent monolayer of cells that express microvilli on the apical membrane, tight junctions between neighboring cells, with various transporters, enzymes and nuclear receptors being present [54], likely explaining the good in vivo prediction of drug absorption [55]. Like Caco-2 cells, since its establishment in 1980 [56], THP-1 cells have been widely used. Compared with other leukemia cell lines [57], THP-1 cells have morphological and functional characteristics similar to human primary monocytes and have a stable gene background, which enhances the reproducibility of results [58]. Finally, besides being biorelevant and well characterized, both cell lines also offer the advantage that they can be easily maintained and manipulated in the laboratory.

## 5. Conclusions

Overall, the remarkable health-promoting effect of SBI was confirmed to relate both to improving intestinal barrier integrity and exerting anti-inflammatory effects, likely originating from the potent gut microbiome modulation by SBI (e.g., production of SCFA and tryptophan metabolites). This work provides the mechanistic underpinning of the health benefits of the medical food in which SBI is the active ingredient (EnteraGam^®^, Proliant Health & Biologicals LLC.). EnteraGam^®^ is currently already used in the dietary management of chronic diarrhea and loose stools resulting from specific intestinal disorders, including irritable bowel syndrome with diarrhea (IBS-D) (based on findings of a clinical study [59]) and inflammatory bowel disease (IBD) [60]. Additionally, SBI is also used to restore the epithelial barrier in subjects with enteropathy [4], while SBI is also already included at lower dosages in dietary supplement products to support immune health. Based upon the modulation of the microbiome and metabolome, there may be new applications for SBI outside of the gut, such as those observed using plasma proteins to provide protection against respiratory infections [61,62,63] along with exerting neuroprotective effects suggesting a potential of plasma proteins in age-associated neurodegeneration [64,65] in preclinical models.

Moreover, the high consistency of findings for SBI and IN with those of clinical studies confirm the predictivity of the SIFR^®^ technology, not only for gut microbiome modulation endpoints [13] but also with respect to the impact on the human host in terms of barrier integrity and immune modulation.

Finally, the study also highlights the potential of the SIFR^®^ technology to, upon inclusion of sufficient test subjects, stratify treatment responses to support future personalized nutrition approaches.

## Data Availability

The datasets generated during and/or analyzed during the current study are available from the corresponding author upon reasonable request. The datasets presented in this article are not readily available due to privacy and ethical restrictions.

## Supplementary Materials

The following supporting information can be downloaded at: https://www.mdpi.com/article/10.3390/nu16111585/s1, Figure S1: Immune modulation was additionally assessed via the production of (a) IL-10, (b) IL1-β, (c) IL-6 and (d) IL-8; Figure S2: SBI increased (a) bacterial cell density (cells/mL) of the gut microbiome of 24 human adults. This originated from a selective increase in specific gut microbes, illustrated by the decreased (b) species evenness (Shannon diversity index); Table S1: Estimates of the linear mixed models for all features.
