# Supplementary material for: Serum-Derived Bovine Immunoglobulin Promotes Barrier Integrity and Lowers Inflammation for 24 Human Adults Ex Vivo
Source: Nutrients. 2024 May 23;16(11):1585. doi: 10.3390/nu16111585 (PMC11174680; doi:10.3390/nu16111585)
Supplement: Supplementary file 1 [file nutrients-16-01585-s001.zip › P0114_supplementary figures.pdf]

nutrients

MDPI

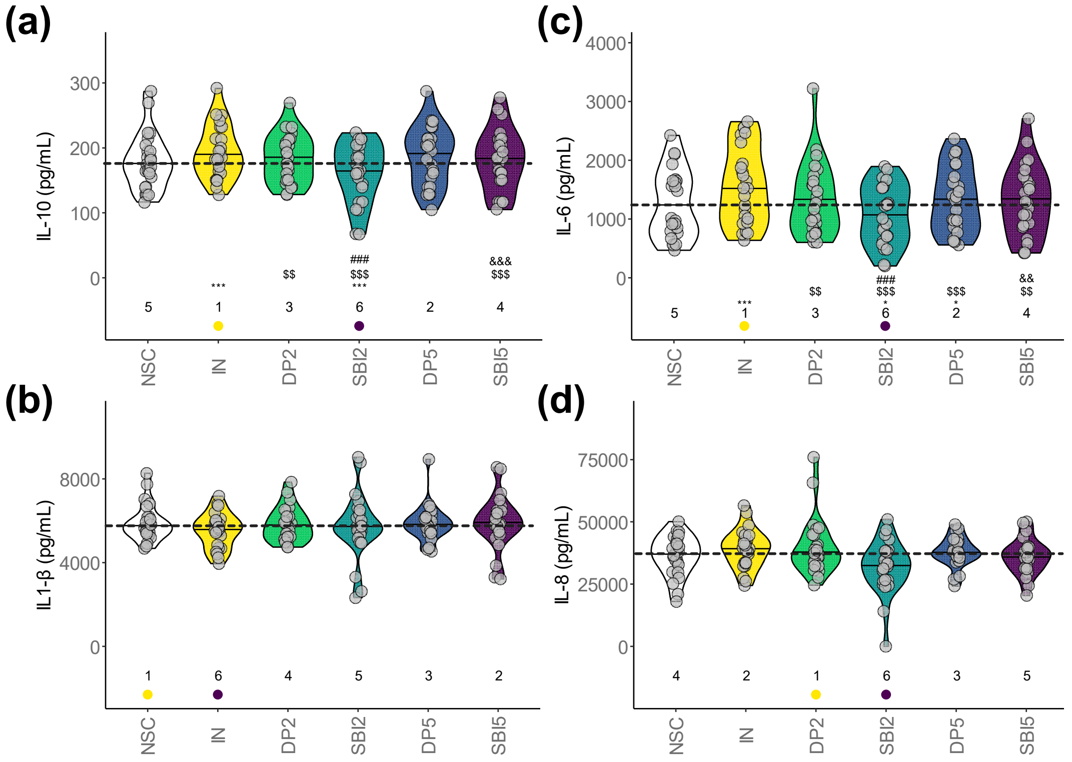

Figure S1. Immune modulation was additionally assessed via the production of (a) IL-10, (b) IL1-β, (c) IL-6 and (d) IL-8. Statistical differences with the NSC are visualized via * ($0.01 < p_{adjusted} < 0.05$), ** ($0.001 < p_{adjusted} < 0.01$) or *** ($p_{adjusted} < 0.001$), '$/$$/$$$' indicate differences with IN, '#/##/###' between corresponding doses of SBI and DP, and '&/&&/&&&' between SBI2 and SBI5 (0.01–0.05/0.001–0.01/<0.001). Ranks of average values per study arm are shown, with lowest/highest values being indicated in purple/yellow, respectively.

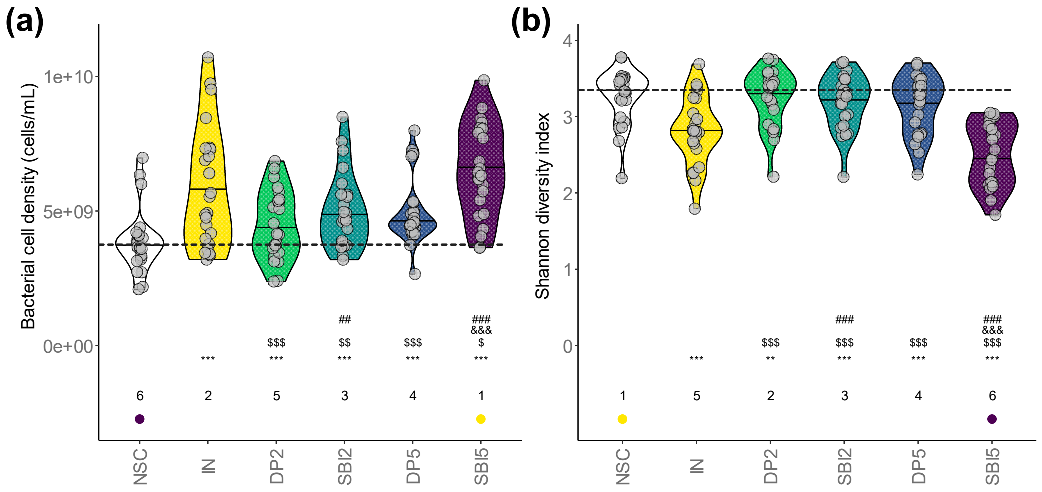

Figure S2. SBI increased (a) bacterial cell density (cells/mL) of the gut microbiome of 24 human adults. This originated from a selective increase of specific gut microbes, illustrated by the decreased (b) species evenness (Shannon diversity index). Statistical differences with the unsupplemented control NSC are visualized via * ($0.01 < p_{adjusted} < 0.05$), ** ($0.001 < p_{adjusted} < 0.01$) or *** ($p_{adjusted} < 0.001$), '$/$$/$$$' indicate differences with the reference prebiotic IN, and '#/##/###' between corresponding doses of SBI and the reference protein DP, and '&/&&/&&&' between the two doses of SBI (SBI2 and SBI5) (0.01–0.05/0.001–0.01/<0.001). Ranks of average values per study arm are shown, with lowest/highest values being indicated in purple/yellow, respectively.
